# Challenges and Prospects of Using Novel Nonlinear Effects in Multimode Optical Fibers for Multiphoton Endomicroscopy

**DOI:** 10.3390/diagnostics16030438

**Published:** 2026-02-01

**Authors:** Lidiya V. Boldyreva, Denis S. Kharenko, Kirill V. Serebrennikov, Anna A. Evtushenko, Viktor V. Shloma, Daba A. Radnatarov, Alexandr V. Dostovalov, Zhibzema E. Munkueva, Oleg S. Sidelnikov, Igor S. Chekhovskoy, Kirill S. Raspopin, Mikhail D. Gervaziev, Stefan Wabnitz

**Affiliations:** 1 Department of Physics, Novosibirsk State University, 1 Pirogova St., 630090 Novosibirsk, Russia; 2 Scientific Research Institute of Neurosciences and Medicine, 4 Timakova St., 630117 Novosibirsk, Russia; 3 Institute of Molecular and Cellular Biology SB RAS, 8/2 Ac. Lavrentiev Ave., 630090 Novosibirsk, Russia; 4 Institute of Automation and Electrometry SB RAS, 1 Ac. Koptyug Ave., 630090 Novosibirsk, Russia; 5 Federal Research Center for Information and Computational Technologies, 630090 Novosibirsk, Russia; 6 Department of Information Engineering, Electronics and Telecommunications (DIET), Sapienza University of Rome, Via Eudossiana 18, 00184 Rome, Italy

**Keywords:** inflammatory bowel disease, colorectal cancer, nonlinear imaging, multiphoton endomicroscopy, multimode fibers, numerical simulations, artificial intelligence

## Abstract

Multiphoton endomicroscopy (MPEM) has recently become a key development in optical biomedical diagnostics, providing histologically relevant in vivo images that are eliminating both the need for tissue damage during biopsy sampling and the need for dye injections. Due to its ability to visualize structures at the epithelial, extracellular matrix, and subcellular levels, MPEM offers a promising diagnostic method for precancerous conditions and early forms of gastrointestinal (GI) cancer. The high specificity of multiphoton signals—the two-photon fluorescence response of endogenous fluorophores (NADH, FAD), the second-harmonic generation signal from collagen, and others—makes this method a promising alternative to both traditional histology and confocal endoscopy, enabling real-time assessment of metabolic status, intestinal epithelial cell status, and stromal remodeling. Despite the promising prospects of multiphoton microscopy, its practical implementation is progressing extremely slowly. The main factors here include the difficulty of delivering ultrashort pulses with high peak power, which is necessary for multiphoton excitation (MPE), and obtaining these pulses at the required wavelengths to activate the autofluorescence mechanism. One of the most promising solutions is the use of specialized multimode optical fibers that can both induce beam self-cleaning (BSC), which allows for the formation of a stable beam profile close to the fundamental mode, and significantly broaden the optical spectrum, which can ultimately cover the entire region of interest. This review presents the biophysical foundations of multiphoton microscopy of GI tissue, existing endoscopic architectures for MPE, and an analysis of the potential for using novel nonlinear effects in multimode optical fibers, such as the BSC effect and supercontinuum generation. It is concluded that the use of optical fibers in which the listed effects are realized in the tracts of multiphoton endomicroscopes can become a key step in the creation of a new generation of high-resolution instruments for the early detection of malignant neoplasms of the GI tract.

## 1. Introduction

Gastrointestinal tract (GI) diseases are very common, with millions of people worldwide seeking medical care for symptoms such as abdominal pain, nausea, and diarrhea. These conditions contribute significantly to overall morbidity and healthcare burden, with a large number of surgical procedures related to the GI. Factors such as infections, lifestyle, and genetic predisposition can trigger GI disorders, which range from functional issues such as Irritable Bowel Syndrome to Inflammatory Bowel Disease (IBD) and cancers. Currently, IBD is diagnosed in approximately 7 million people worldwide [1,2,3]. Patients with IBD have been shown to have an increased risk of developing colorectal cancer (CRC) [4,5,6]. IBD includes a spectrum of chronic inflammatory GI pathologies, two major subtypes of which are ulcerative colitis and Crohn’s disease, which have the highest risks of cancer progression [7]. Ulcerative colitis affects the colon and rectum primarily and is characterized by mucosal damage, which is followed by cryptitis and crypt abscesses. Crohn’s disease is characterized by transmural inflammation, noncaseating granulomas, and a thickened submucosa in the small and large intestine, and may also affect the mouth, esophagus, stomach, and anus [1]. IBD remains a chronic relapsing disorder with complex etiology. The known risk factors for IBD include genetic predisposition, environmental stress, diet, and intestinal microbiota composition [8,9]. The impact of heritable factors was identified in human studies, in which first-degree relatives of IBD patients were predisposed to intestinal inflammation at a higher rate than the general population [9,10,11]. More than 200 genetic loci were identified as IBD-linked, including major inflammatory genes that may promote an exaggerated immune response in the intestine [12]. However, the onset of the disease, its characteristics, and its severity can be modulated by additional risk factors like a Western diet or smoking and alcohol abuse [13,14]. Despite significant progress in understanding the etiology and risk factors for IBD and CRC as well as great improvement in therapeutic strategies, 40–70% of patients require surgery [15].

The human intestinal epithelium, with its main metabolic and digestive functions, represents approximately 400 m^2^ of the rapidly proliferating monolayer with a complete turnover of 24 to 96 h [16]. The proliferative compartment of the epithelium is localized at the bottom of an intestinal crypt, followed by a gradient of increasingly differentiated epithelial cells along the vertical axis [17]. Another one of the main intestinal epithelium functions is to coordinate appropriate immune responses, including tolerance and pathogen-specific immunity, via innate and adaptive immune systems [18]. A healthy intestinal epithelium layer provides an efficient physical, chemical, and electrical barrier against a luminal microbial community and chemical factors. The intestinal epithelial barrier includes three key levels, pre-epithelial, epithelial, and post-epithelial defense, the latter being represented by the lamina propria [19]. The pre-epithelial barrier is composed of the mucus glycoproteins associated with other proteins and lipids and a bicarbonate-rich compound of the mucus, maintaining a neutralizing pH at the epithelial surface. Once the inflammatory response in the intestine has started in IBD, Tumor Necrosis Factor alpha (TNF-α), Interleukin one beta (IL-1β), and other inflammatory factors amplify the immune reaction, leading to further damage of the intestinal mucosa [20]. Following this inflammation, the integrity of the epithelium is lost, and luminal antigens, including pathobionts, invade the subepithelial compartment, thus activating and/or sustaining deregulated inflammatory immune responses [21,22]. One of the prominent features of IBD is recurrent inflammation [19] that can not be resolved by innate anti-inflammatory and wound-healing mechanisms. Long-term inflammation finally develops into a chronic form and results in tissue remodeling and substantial modifications in cell physiology and metabolism, increasing the risk of CRC development [23,24]. Precise diagnosis of these GI diseases is still difficult and time-consuming, although early and accurate diagnostics, especially regarding oncological cases, are ultimately needed for timely and successful cures. Non-invasive diagnostic methods could eliminate the need for physical biopsy and provide improved diagnostic accuracy for a range of GI disorders. Despite the accessibility of imaging techniques that are exploited in combination with standard WLE (white light endoscopy) in clinical practice, such as narrow band imaging (NBI) [25], autofluorescence imaging (AFI) [26], and confocal endomicroscopy (CLE) [27,28], there is a lack of accurate methods for detecting precancerous lesions of the GI tract. Moreover, there is currently no accessible in vivo technology that can reliably indicate if a biopsy should not be performed. Despite the use of advanced imaging techniques, multiple biopsies are still required in most cases to confirm or refute an endoscopic diagnosis.

The development of optical technologies has made a significant contribution to the expansion of the capabilities of endoscopic analysis and has given rise to the use of new generations of instruments for the endoscopy and examination of GI biopsies. The spectra of the current endoscopic image enhancement techniques include narrow band imaging (NBI), compound band imaging (CBI), i-SCAN optical enhancement, linked color imaging (LCI), flexible spectral imaging color enhancement (FICE), hemoglobin enhancement (HbE), red dichromatic imaging (RDI), and blue light imaging (BLI) [29,30]. Other techniques in various phases of development include autofluorescence imaging, endocytoscopy, confocal laser endomicroscopy, optical coherence tomography, multiphoton microscopy, and Raman spectroscopy [31]. Confocal microscopy techniques for GI biopsies in recent decades have advanced from laboratory tools to “optical biopsy” methods called confocal laser endomicroscopy (CLE), which also provide real-time, in vivo cellular visualization during endoscopy [29,32]. These technologies, including both endoscope-based and probe-based systems, usually use fluorescent labeling and contrast agents to achieve high-resolution images, assess tissue structures, and identify lesions, and only partially substitute for the clinical use of traditional biopsies and WLE [33]. Besides the limited availability of certain laser wavelengths and the need for tissue staining procedures in biopsy or contrast agent infusion, CLE limitations include a high energy load on tissue.

Multiphoton microscopy (MPM) offers undoubted advantages over other optical microscopic imaging techniques in terms of penetration depth and reduced photobleaching, which has allowed it to gain widespread use over the past 30 years [34] for the in-depth study of neurons, tumors, cancer, and other types of biological tissue [35,36]. This method utilizes excitation by the absorption of two or more photons in a single quantum event. Since the photon energy is inversely proportional to its wavelength, photons with twice the wavelength are required. Generating a significant number of multiphoton excitation events requires a well-focused laser beam. As a result, fluorophore excitation occurs only in a small volume in the focal plane, significantly reducing photobleaching and UV-induced light damage in live-cell imaging and enabling three-dimensional imaging of tissue structure [37]. Multiphoton imaging technology also provides deeper light penetration (more than 700 µm) and lower background signal. Excitation light sources for such spectroscopy typically operate at wavelengths from 650 nm to 1064 nm, but using water transparency windows near 1300 nm or 1700 nm, it is possible to obtain a signal and construct deeper nonlinear images for two-photon and three-photon cases, respectively [38]. Furthermore, high-energy (tens of nJ) femtosecond or picosecond pulses with a relatively high repetition rate are required to achieve a sufficient imaging speed. The optimal value is approximately 10 MHz. At higher frequencies (approximately 100 MHz), the average radiation power becomes extremely high, leading to sample overheating, while low frequencies (100 kHz) proportionally (by two orders of magnitude) reduce the scanning time.

The real challenge in recent years has been to move from an expensive standard bulky multiphoton microscopy setup to an endoscopic format, which will allow intravital and clinical patient imaging [39,40,41]. Special optical fibers play a crucial role for this technology. Such fibers can act both as a waveguide of intensive ultrashort laser pulses [42,43], which are necessary for the multiphoton fluorescence excitation, and as a nonlinear element, significantly broadening the spectrum of the initial radiation [44,45] and allowing the implementation of such approaches as multiplexed coherent anti-Stokes Raman scattering (M-CARS) [46,47] and optical coherence hyperspectral [48] microscopy.

In this narrative review, we look through the fundamental optical properties of live tissues in the context of inflammation assessment, detection of GI tissue pathological remodeling, and demarcating pathological and tumor regions from healthy ones. We attempt to find out the possible requirements for an MPEM that utilizes novel nonlinear effects in optical fibers like Kerr self-cleaning and supercontinuum generation to improve delivery of ultrashort laser pulses. By approaching this problem comprehensively—from the perspective of the physics of nonlinear processes in multimode and special fibers, engineering limitations of optical system miniaturization, clinical application scenarios, fluorescence properties of a tissue, and biological criteria for the early diagnosis of gastrointestinal disease—we aim to outline the parameters that determine the practical feasibility and biomedical value of the next generation of MPEM systems. To do this, we performed a structured literature search using the PubMed, Scopus, Google Scholar, and ResearchGate databases through November 2025, focusing on research articles, meta-analyses, guideline statements, key translational studies, and randomized controlled trials. Search terms included but were not limited to “optical imaging endoscopy”, “multiphoton endoscopy”, “hollow-core fibre”, “confocal laser endomicroscopy”, “narrow-band imaging”, “nonlinear imaging”, “multimode fibers”, “fluorescence endoscopy”, “Raman spectroscopy”, “gastrointestinal”, and “IBD”. Additional studies were collected from bibliographies of major reviews. To enhance understanding of practical application relevance of the described technologies for clinical use, we prioritized evidence stratified by experimental and clinical indication. As a result, this kind of interdisciplinary analysis allowed us to identify the key conditions necessary for integrating complex nonlinear phenomena in optical fibers into clinically oriented MPEM.

## 2. Optical Properties of Healthy and Diseased GI Tissues and Detection Methods

Absorption and scattering properties of the biological tissue are the key parameters involved in any optical-based methods and approaches for medical diagnostics and treatment, including CRC detection. On the one hand, these parameters define interaction and scattering of light incident on the tissue, so the knowledge of their values is vital for the development of direct laser-based approaches for medical treatment and diagnostics. Moreover, the knowledge of these parameters is necessary for the development of synthetic phantoms of biological tissue with close values of the absorption and scattering parameters. Such phantoms would have a similar response to incident radiation, which is necessary for testing various medical devices and in scientific research. On the other hand, these parameters themselves can be used for CRC detection, enabling non-invasive tissue identification and tumor delineation in real time.

**Figure 1:**
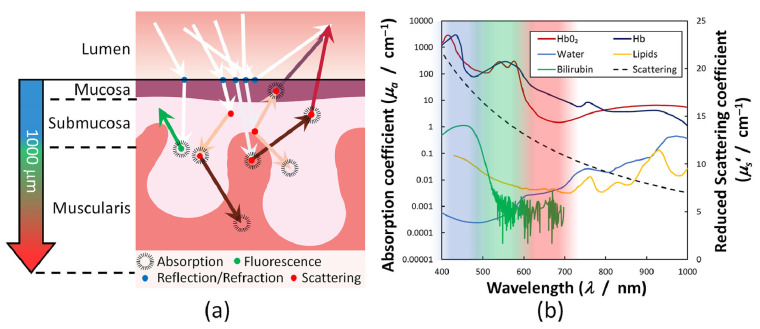
Scheme of the main physical processes in the light–tissue interaction under optical-based medical diagnostics and treatment (**a**). Optical properties (absorption and reduced scattering coefficient) of key specific molecules such as oxygenated (HbO_2_) and deoxygenated (Hb) hemoglobin, lipids, bilirubin, and water (**b**) (from Clancy et al., used under CC-BY 4.0 license [50]).

Specifically, when light illuminates multi-layered bowel tissue (see Figure 1a), it is partially reflected at the surface interfaces with refractive index mismatches, and penetrates into the tissue, where processes of scattering and absorption may occur. Through multiple scattering events, a fraction of this light eventually re-emerges and can be measured for various diagnostic techniques. Moreover, some molecules may also exhibit autofluorescence, which will be described in the following chapters. To characterize light absorption in tissues where it significantly dominates over scattering, the Bouguer–Beer–Lambert law provides an accurate model for light attenuation [49]:

$$I\left(\lambda \right)=I_{0}\left(\lambda \right)\exp\left(-\mu_{a}\left(\lambda \right)\times d\right)$$

where $I_{0}\left(\lambda \right)$ is an incident light intensity, *d* is the sample thickness, and $\mu_{a}\left(\lambda \right)$ is the spectral dependent absorption coefficient that can be calculated from the measured total transmittance $T_{t}\left(\lambda \right)$ and total reflectance $R_{t}\left(\lambda \right)$ spectra from a sample as follows: $\mu_{a}\left(\lambda \right)=\left(1-\left(T_{t}\left(\lambda \right)+R_{t}\left(\lambda \right)\right)\right)/d$. The absorption coefficients of key specific molecules such as oxygenated (HbO_2_) and deoxygenated (Hb) hemoglobin, lipids, bilirubin, and water are presented in Figure 1b, revealing the predominance of hemoglobin and bilirubin in the visible spectral range, where water absorption is negligible. In contrast, in the near-IR spectral region, water has a higher absorption coefficient along with lipids. The total absorption coefficient $\mu_{a\;\mathrm{total}}\left(\lambda \right)$ of the biological tissue represents a sum of the values of this coefficient for distinct biomolecules $\mu_{ai}\left(\lambda \right)$, taking into account their concentrations $f_{i}$ as follows: $\mu_{a\;\mathrm{total}}\left(\lambda \right)=\sum \mu_{a\;i}\left(\lambda \right)\times f_{i}$.

The scattering-based tissue parameters include the reduced scattering amplitude $\alpha^{*}$, Mie scattering power $b_{\mathrm{Mie}}$, the percentage contribution of Rayleigh scattering $f_{\mathrm{Ray}}$, and the anisotropy factor of *g*. All together, these define the scattering coefficient $\mu_{s}$ and the reduced scattering coefficient $\mu_{s}^{*}$ as follows [51]:

$$\mu_{s}^{*\mathrm{total}}=\mu_{s}^{\mathrm{total}}\left(1-g\right)=\alpha^{*}\left[\left(1-f_{\mathrm{Ray}}\right){\left(\frac{\lambda }{\lambda_{0}}\right)}^{-b_{\mathrm{Mie}}}+f_{\mathrm{Ray}}{\left(\frac{\lambda }{\lambda_{0}}\right)}^{-4}\right].$$

The scattering curve presented in Figure 1b, based on typical bowel tissue values, reveals that scattering decreases at longer wavelengths. The scattering properties of tissue are determined by its microstructure, arising from extra- and intra-cellular inhomogeneities. These include variations in particle size and refractive index mismatches at multiple scales, from specific molecules like collagen fibers to organelles like mitochondria and cell membranes. The following are the most common methods for measuring the optical properties of biological tissues.

**Figure 2:**
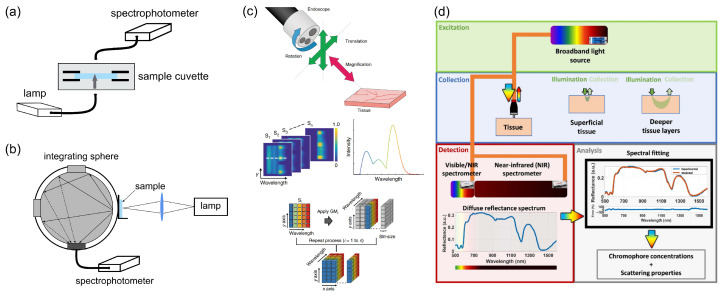
(**a**) Collimated transmittance measurement setup (from Carneiro et al., used under CC-BY 4.0 license [53]). (**b**) Total transmittance setup based on an integrating sphere (from Shahin et al., used under CC-BY 4.0 license [58]). (**c**) Hyperspectral measurement setup and the principles of tissue’s optical properties characterization (from Yoon et al., used under CC-BY 4.0 license [57]). (**d**) Scheme of Diffuse Reflectance Spectroscopy and the principles of tissue’s optical property characterization based on this technique (from Nogueira et al., used under CC-BY 4.0 license [51]).

Conventional spectral measurements (see Figure 2a,b) provide an ex vivo approach for characterizing the optical properties of colorectal tissues. In this approach, a light source with a broad wavelength range, covering the visible and near-infrared spectrum, is used in conjunction with a spectrophotometer. Key optical parameters such as total transmittance $T_{t}\left(\lambda \right)$, total reflectance $R_{t}\left(\lambda \right)$, and collimated transmittance $T_{c}\left(\lambda \right)$ are collected by an integrating sphere [52] for diffuse light or using a collimated transmittance setup [53]. These experimental spectral measurements serve as the input for analytical models of light propagation in turbid media. Techniques (e.g., the inverse adding-doubling method [54], the inverse Monte Carlo method [55], etc.) are employed to extract optical parameters, including $\mu_{a}\left(\lambda \right)$, $\mu_{s}\left(\lambda \right)$, and $\mu_{s}^{*}\left(\lambda \right)$. Once calculated, these parameters enable quantitative comparisons between healthy and diseased tissues and facilitate the development of accurate tissue-simulating phantoms for biophotonic applications.

Hyperspectral analysis (see Figure 2c) enables ex vivo and in vivo characterization of tissue optical properties. It employs a xenon or halogen lamp as a light source and a hyperspectral camera that collects spectral information from each pixel of a captured image [56,57]. The hyperspectral camera can be integrated into the endoscopic observation module for real-time diagnostics of colorectal cancer. In this approach, a two-dimensional collected dataset, referred to as “hyperspectral data,” is transformed from reflection spectra into absorption spectra. However, the presence of undulation on the human tissue surface results in hyperspectral data dependencies from the distance and angle of each measurement point that lead to the need to normalize the spectral data.

Diffuse reflectance spectroscopy (DRS) is another approach for non-invasive tissue diagnostics and optical parameter measurements based on illuminating a tissue sample with a light source in the visible or near-infrared range and analyzing the resulting diffuse reflectance of light propagating inside the tissue (as presented in Figure 2d). As was mentioned above, the reflected spectrum is quantitatively related to the tissue’s optical properties, namely its absorption and scattering coefficients, which serve as biomarkers for its physiological and structural state. Fitting the measured diffuse reflectance spectra based on reflectance values obtained by different approaches (e.g., Monte Carlo simulation of light propagation in tissues) enables reconstruction of these optical parameters and therefore the tissue chromophore concentrations in the target tissues. The latter can be used for medical diagnostics, including cancer detection, because mucosa and tumor tissues have different concentrations of these biomolecules. However, colorectal cancer detection using DRS techniques faces some constraints related to the limited depth below the tissue surface for measurements, specific wavelength range for detection of certain biomolecules, large numbers of required spectra to be measured, and limitations of analytical models in describing all cases.

As was mentioned in the introduction part, chromophore concentrations (total hemoglobin, lipid, water, bile, and met-hemoglobin) and tissue scattering parameters (Mie scattering power, scattering amplitude, and percentage contribution of Rayleigh scattering) can be used for CRC detection, enabling non-invasive tissue identification and tumor delineation in real time.

In particular, it was found that tumors had larger concentrations of total hemoglobin and water, a smaller concentration of lipids, and a lower level of tissue oxygen saturation (StO_2_) compared to normal mucosa [51]. In total, hemoglobin concentrations of 13.6±8.8 mg/dL and 72.0±29.2 mg/dL were measured for normal mucosa and adenomatous polyp, respectively [59]. The same dependence on an increase in total hemoglobin concentrations was observed in [60], where total hemoglobin concentration increased from 93.4±17.1 µM for normal tissue to 153.8±38.6 µM for tumors.

Moreover, the scattering properties of tumors differ from normal tissue, showing a lower fraction of Rayleigh scattering ($f_{\mathrm{Ray}}$) and higher Mie scattering power ($b_{\mathrm{Mie}}$) and amplitude of reduced scattering coefficient $\alpha^{*}$ for tumors in comparison with normal mucosa [51]. Specifically, the Mie scattering power increases from 0.2±0.2 for normal mucosa to 0.5±0.3 for tumor, whereas the amplitude of the reduced scattering coefficient $\alpha^{*}$ increases from 8.2±3.7 for mucosa to 12.0±5.6 for tumor tissue. Taking into account these differences and the combination of the parameters enables an efficient classification of normal mucosa and cancer tissues with an accuracy of 88.3%±0.6%.

## 3. The Use of Autofluorescence Approaches for GI Diagnostics

Many of the modern endoscopic methods to obtain images of the GI tissues traditionally use special dyes, such as fluorescein, cresyl violet, and acriflavine. The first one stains the intercellular space, surface epithelial cells, and lamina of the mucous membrane. The second one (cresyl violet) predominantly stains the cytoplasm and negatively visualizes the nuclei. It is also used for staining nervous tissue, mast cells, and cartilage. Finally, acriflavine stains surface epithelial cells, nuclei, and, to a lesser extent, the cytoplasm. However, these dyes are quite often not harmless to the patient’s body, so recently, the autofluorescent properties of the body’s own molecules, such as certain coenzymes (e.g., NADH), stromal structural proteins (collagen, elastin), and endogenous porphyrins [61], have begun to be used for the diagnosis of GI diseases. Numerous intracellular molecules have autofluorescent properties with identifiable excitation and emission spectra. Contributing natural fluorophores include the metabolic coenzymes nicotinamide adenine dinucleotide phosphate (NAD(P)H) and flavin adenine dinucleotide (FAD) [62,63], structural collagen family proteins, and heme proteins such as cytochrome c and protoporphyrin IX, as well as a lysosomal product (pigment)—lipofuscin [64,65,66,67]. For most of these molecules, UV or visible light results in the greatest absorption of light and, consequently, the greatest fluorescence emission. It should be noted that mitochondria and lysosomes are the intracellular structures that produce the majority of autofluorescence in living tissue.

Spectral assessment of cellular autofluorescence is a powerful tool for identifying cell types and their status because endogenous fluorophores have unique spectral signatures—excitation and emission wavelengths (see Table 1)—that reflect the metabolic state, morphology, and health of cells and tissues. Successful applications of autofluorescence assessment include tumor surgical margin determination [68,69,70], assessment of the effects of age and drug exposure on stem cells [71], and specific cell properties such as reactive oxygen species (ROS) levels and cell cycle stage [72,73,74].

Autofluorescence spectroscopy (AFS) is one of the new analytical methods based on the assessment of endogenous fluorophores in tissues and biological fluids for diagnostics [91]. The major limitation in the use of autofluorescence for differentiating healthy and pathological tissue areas is that autofluorescence measuring is currently available only at the upper layers of cells. Cancer and other pathophysiological processes may alter the amount and distribution of endogenous fluorophores in tissue [92,93]. Such changes can be found by using autofluorescence microscopy. This shows that differences in the clinically measured autofluorescence spectra between normal and cancerous colonic tissue are mainly due to thickening of the tumor mucosa, resulting in a reduced submucosa fluorescence contribution, as well as increased hemoglobin absorption in tumor tissue [93]. The advantage of using autofluorescence to demarcate cancerous areas is that there is no need for any exogenous substances for the endoscopy and no staining procedures for biopsy samples. Several studies have demonstrated the autofluorescence aptitude to distinguish between healthy and cancerous tissue in patients, in particular for CRC [94,95], oral neoplasms [96], skin cancer [97], and cervical lesions [98]. The results of these studies show that autofluorescence signals between normal and cancerous tissue differ and can be used for non-invasive diagnosis and tumor margin detection. A number of previous studies have focused on differences in autofluorescence emission spectra between healthy and diseased colonic tissue. These studies have demonstrated differences between a variety of tissue types, including the following cases: normal tissue and adenomatous (i.e., neoplastic) polyps ex vivo [99,100]; normal tissue and adenomas in vivo [101]; normal tissue or hyperplastic polyps and adenomas [102]; hyperplastic polyps and adenomas [103]; and all three of the above tissue groups among themselves [104]. Cancers of the upper gastrointestinal tract (esophagus and stomach) have also been investigated [105].

An important indicator of autofluorescence is the fluorescence lifetime [106], which differs in normal and diseased tissue. Coda et al. [107] demonstrated differences in fluorescence lifetimes between healthy and neoplastic tissue in ex vivo experiments. Furthermore, an increase in autofluorescence lifetime was found in IBD compared to healthy gut tissue [107]. In a recent study, Herrando et al. assessed the intravital autofluorescence properties of colorectal tissues [76]. Collagen types I-IV, NAD(P)H, and flavins (FAD), which are the dominant tissue fluorophores in the colorectal mucosa, were selected as endogenous fluorophores. The author’s results provide compelling evidence that multiparametric autofluorescence lifetime measurements can provide additional functional and structural characterization of the tumor and adjacent normal (healthy) areas, potentially aiding in clinical decision-making in both diagnosis and treatment.

## 4. Modern GI Histology and Ex Vivo Models

The modern endoscopy arsenal uses forefront optical methods, which have significantly improved the detailed assessment of neoplastic changes in the GI mucosa, especially for biopsy-related applications. However, performing either both standard and multiple biopsies or endoscopic mucosal resection for extended biopsy is associated with the risk of complications (bleeding, perforation). Moreover, frequent false-negative results, along with the expensive, labor-intensive, and multi-stage process of histological analysis, lead to significant material and time costs. One important rapidly developing area is the creation of ex vivo technologies, biobanking of biopsies, 3D culture, and organoids [108,109,110]. The ex vivo cultivation of biopsies has opened up great possibilities of thorough microscopic examination of diseased tissue samples without the need for multiple interventions [111,112]. While these approaches significantly advanced the study of the molecular mechanisms of GI pathologies and also gave rise to achieving detailed data for personalized therapies, they do not compensate for the need to examine the impaired areas of the GI mucosa in real time. Intravital microscopy (IVM) was initially developed to visualize biological processes in living organisms at a cellular level, overcoming the limitations of traditional microscopy that formerly required fixed tissue biopsy samples. Modern IVM has advanced significantly with the integration of fluorescence microscopy, two-photon microscopy, and nonlinear microscopy [113]. In the last decades, IVM has been aimed at developing the direct, real-time observation of tissues at a cellular and subcellular resolution in a minimally invasive way and has especially been oriented toward being implemented in human clinical settings. The approaches referred to as optical biopsy cover IVM scanning to examine tissues in vivo, visualizing fluorescently labeled molecular markers, and use of natural fluorophores to examine the acute immune response activation, such as leukocyte extravasation, as far as chronic inflammation, tumor growth and metastasis, vascularization, and metabolic shift in cellular processes.

The development of non-invasive and rapid diagnostics for GI diseases has been an extremely high-priority area of research.

**Figure 3:**
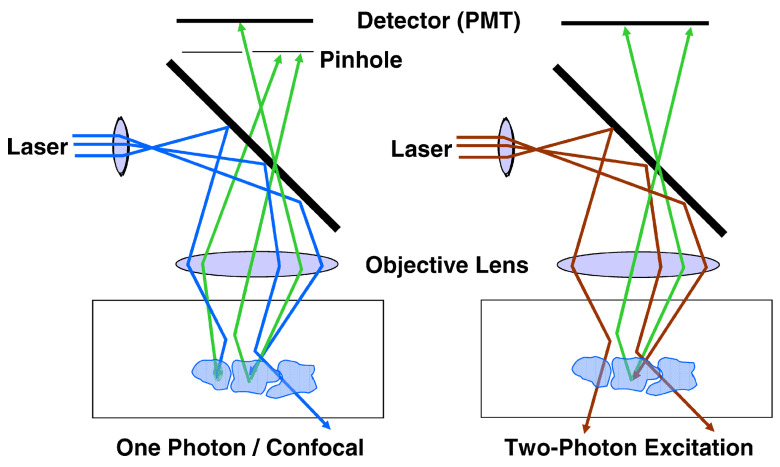
Effect of scattering in confocal microscopy (**left**) and two-photon excitation microscopy (**right**) (from D. W. Piston, used under CC-BY license [115]). The arrows indicate the excitation (UV and IR wavelengths are shown in blue and brown, respectively) and emission (green) pathways.

Although the concept of confocal microscopy was developed by M. Minsky much earlier [114], it was only the emergence of high-speed computing systems in the late 1990s that made possible the creation of a new technology—confocal laser microscopy (see Figure 3 left). This was followed by the development of confocal laser endomicroscopy (CLE). CLE is a minimally invasive technique that uses a miniature confocal endomicroscope built into an endoscope to obtain high-resolution microscopic images of the GI mucosa in vivo. Such endomicroscopes typically use continuous-wave laser radiation with wavelengths ranging from 480 to 488 nm; less frequently, other wavelengths are also used, such as 568 nm and 633 nm. Two different CLE systems have been used in clinical endoscopy—endoscope-based CLE (eCLE) and probe-based CLE (pCLE).

With eCLE, the confocal probe is directly integrated into the tip of a conventional endoscope (Pentax EG-3870CIK and Pentax EC-3870CLICK, Tokio, Japan) [116]. An eCLE system combines conventional endoscopy with CLE, eliminating the need for a separate device for routine exams of the upper and lower GI tracts. Such an integrated system provides high magnification and is considered a powerful but expensive tool for detecting lesions in the esophagus, stomach, and colon. It uses low-power laser sources at different wavelengths to illuminate the tissue and detect the fluorescent light that bounces back from the cells. The “confocal” aspect ensures that only light from a specific focal plane passes through, creating a high-resolution image of the cellular structure. A fluorescent contrast agent, such as fluorescein sodium, is typically administered intravenously to highlight the tissue structures. Images are acquired from the surface of the lens down to a depth of about 250 microns. However, there are some disadvantages of the eCLE method, e.g., a big size of the eCLE endoscope can make intubation difficult, especially in the upper GI tract. Another disadvantage of eCLE is the high cost of such systems. Therefore, some eCLE systems are no longer commercially available [117].

With probe-based CLE (pCLE), a laser-scanning microscope is integrated into a slender probe that can be passed through the channel of a standard endoscope. The probe is placed on or in contact with the tissue of interest. A low-energy laser is transmitted through the probe, illuminating the tissue. The reflected light is sent back through the same probe, allowing the system to create an image at the cellular or even subcellular level. Fluorescent dyes, such as fluorescein, are often used to enhance contrast and visualize the cellular structures. The device can be delivered in different ways: (I) into the luminal gastrointestinal tract through the working channel of standard endoscopes; (II) into extraluminal cystic and solid parenchymal lesions through an endoscopic ultrasound (EUS) needle; or (III) into the biliary system through an endoscopic retrograde cholangiopancreatography (ERCP) accessory channel (Cellvizio, Mauna Kea Technologies, Paris, France) [118].

Key applications and benefits of pCLE include real-time diagnostics, which provide immediate microscopic information to help diagnose lesions and differentiate between benign and malignant tissues. Improved biopsy guidance allows clinicians to guide biopsies to the most relevant part of a lesion, increasing the yield and accuracy of the procedure and reducing the need for biopsies. In some cases, pCLE can provide enough diagnostic information to avoid the need for a traditional biopsy, saving time and reducing costs. Furthermore, pCLE has good versatility: different probes can be used depending on the procedure, such as specialized probes for the biliary tract (e.g., CholangioFlex) or the gastrointestinal tract (e.g., GastroFlex) [119].

Of course, pCLE has some limitations in comparison with eCLE, such as limited depth of imaging, only single-plane imaging, and a resolution that may be slightly lower than other methods like endomicroscopy, performed with dedicated endoscopes.

## 5. Multiphoton Microscopy for In Vivo Imaging

**Figure 4:**
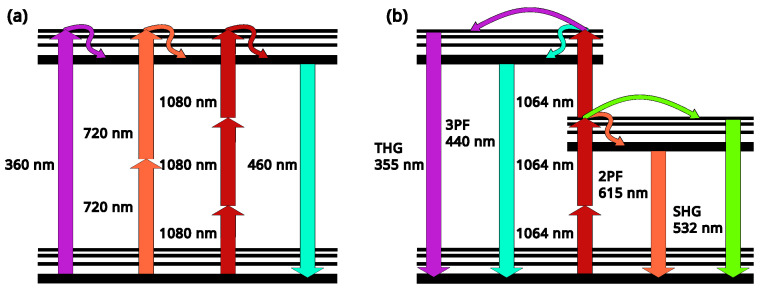
Schematic representation of the energy levels of the fluorescence process: (**a**) an example of a fluorescent dye that emits light at 460 nm. One (purple), two (orange), or three (red) photons are absorbed to emit a photon of fluorescence (turquoise) (reworked from A. Diaspro, redrawn under CC-BY license [127]). (**b**) An example of different processes excited by one ultrashort pulse at 1064 nm: third (purple) and second (green) harmonic generation (THG and SHG, respectively); three (3PF, turquoise) and two (2PF, orange) photon fluorescence.

In recent years, MPM technology has become an indispensable imaging tool in tissue research with its submicron spatial resolution, millimeter-level imaging depth (versus 100 microns for confocal microscopy, see Figure 3 right), and three-dimensional tomography capabilities [120]. The most promising application of MPM is imaging of deep scattering tissues, especially in vivo. This allows one to perform measurements with an extremely high resolution and observe neuronal activity, pathological processes in tumors or cancer [121], and the metabolism of a living cell [122,123] in real time. This enables researchers to address key problems in biology and biomedicine, uncover disease mechanisms, and develop new effective drugs and therapeutic approaches. The basic principles of multiphoton processes are depicted in Figure 4. On the left side (Figure 4a), it is shown schematically that the same fluorescence process can be excited by different wavelengths as a result of the absorption of one, two, or three photons. At the same time, pump radiation at one wavelength (e.g., 1064 nm) can activate a few nonlinear processes like harmonic generation and two- and three-photon fluorescence (see Figure 4b) in the case where absorption spectra of the transitions match well with the conditions of the corresponding processes.

It should be noted that activation of such multiphoton processes requires really high-intensity laser beams (peak power of dozens of kilowatts and more), which raises a question about photodamaging, especially for investigation of living cells. On the one hand, the longer wavelengths and the highly localized region in which fluorescence is excited reduce the photodamage of the whole sample in comparison with confocal microscopy [124]. However, on the other hand, deep tissue imaging requires more optical power, which increases the load on a sample in the focal point. There are two general directions to mitigate a negative impact of high intensities of light. The first one is to increase the carrier wavelength even more to reach the so-called “second biological transparency window” [125], especially the regions near 1.3 and 1.7 µm [38]. The second one is to reduce the pulse duration down to a few dozen femtoseconds to achieve low average excitation powers [126]. Both directions are currently in the active research stage, and the question of photodamage in MPEM must be considered in the context of the parameters of a specific experimental setup and the sample being studied.

Nonlinear imaging with ultrashort pulsed lasers has long been a subject of research for intraoperative endoscopy applications due to the lack of a need for toxic specimen staining and their inherent optical sectioning (no need for specimen slicing), among other advantages. To date, a wide range of schemes have been demonstrated that allow single-mode imaging using two-photon [128] and multiphoton fluorescence [40], second and third harmonic generation, and CARS [129]. The scanning area and tissue penetration depth of the listed systems are limited by the mode field diameter and radiation intensity, respectively. In this regard, the use of multimode (MM) fibers in an endoscopic system is promising since they have a larger mode size and support the propagation of radiation with higher intensity. However, the use of MM fibers for imaging was considered to be difficult due to random coupling of transverse modes, especially in the presence of mechanical stresses (e.g., bending). This coupling distorts the information as the pulse propagates, making high-resolution, real-time imaging impossible.

## 6. Advantages and Challenges of Using Optical Fibers

### 6.1. Beam Self-Cleaning and Supercontinuum Generation

In recent years, the situation has changed dramatically, and MM optical fibers are currently experiencing tremendous growth in research interest. This is driven, on the one hand, by the development of sources and means for analyzing laser radiation and, on the other hand, by a significant increase in transmitted signal power, at which standard single-mode fibers become inapplicable. In particular, ambitious tasks are being set again, such as transmitting images through such fibers [130,131]. To solve this problem, some scientific groups use spatial light modulators. In this approach, a preliminary deep learning process is required to determine the fiber transmission matrix that takes into account random intermode coupling [132,133,134,135]. However, another solution also exists and it lies in using nonlinear imaging methods based on MM and Hollow-Core (HC) fibers. MM fibers take advantage of the virtually instantaneous effect of Kerr self-cleaning of the beam (also known as beam self-cleaning, BSC) [136,137], eliminating the need for external modulation systems or training steps that would determine the fiber transfer matrix. HC fibers can just propagate high-power femtosecond pulses with minimal distortions and allow the use of different gases as a nonlinear medium [43]. This happens due to small chromatic dispersion and nonlinearity values as far as the light propagates commonly in the air rather than in glass as occurs in standard fibers. Few attempts have been made to investigate the dispersion curve in such fibers, both experimentally [138] and theoretically [139,140]. Finally, it was found that each transmission window has its own zero dispersion wavelength (ZDW), and the third-order dispersion depends on the core radius, decreasing with increasing radius. So, with a high enough fiber core and a carrier wavelength of ultrashort pulses near to the ZDW, there is no need to worry about pulse pre-compensation at all. At the same time, a low nonlinearity value is also achieved due to a large diameter of the fundamental mode, which dominates due to significantly higher leakage losses on higher-order modes. Moreover, the double-clad design of HC fibers allows the use of the same fiber both for excitation of nonlinear processes and collection of fluorescent light. Some schemes have already demonstrated the applicability of HC [141,142] and a combination of HC and MM gradient index (GRIN) fibers [143] for endoscopic systems where nonlinear visualization is achieved by means of CARS and second harmonic generation.

**Figure 5:**
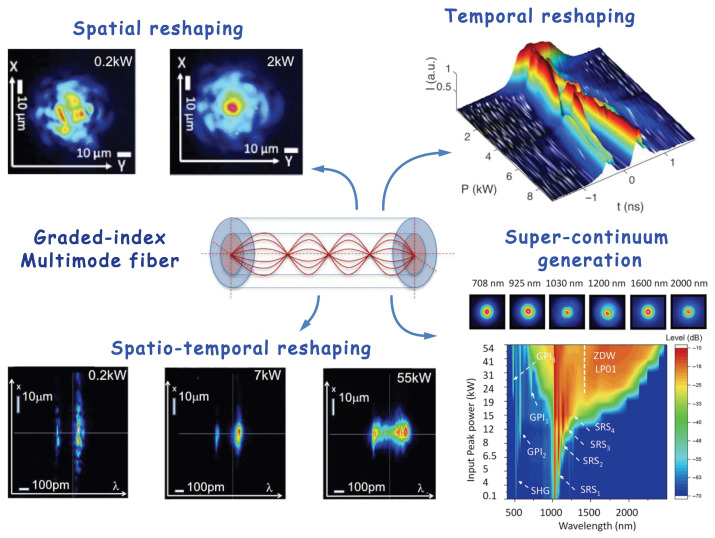
Diversity of nonlinear effects in multimode fibers (adopted from Cristiani et al. and Moussa et al. under CC-BY 4.0 license [137,145]).

BSC is a nonlinear effect occurring at relatively high optical powers (1–10 kW), manifested as follows: the chaotic intensity distribution (speckle) at the fiber output in low-power mode is transformed into a Gaussian-like beam as the power increases [136], while temporal reshaping also occurs (see Figure 5). This phenomenon ensures precise focusing on the sample, contributes to the efficiency of the entire system [137], and provides the high resolution required for tissue structure imaging using multiphoton microscopy [120,144]. This innovation significantly simplifies and speeds up the beam focusing process and reduces the cost of the device itself. The resulting improvement in beam brightness remains resistant to mechanical impact on the fiber, which is especially advantageous for endoscopic visualization systems.

The spatiotemporal characteristics of beam self-cleaning, combined with the accompanying increase in peak pulse power and supercontinuum generation (see Figure 5), open the way for important improvements in nonlinear imaging processes. These include the integration of multiphoton fluorescence with second- and third-harmonics, as well as coherent Raman-based processes such as CARS or stimulated Raman scattering (SRS) [47,137,146]. This novel concept could be based on the nonlinear response of the GRIN MM fiber medium to the propagation of high-peak-power optical pulses in combination with precise control of the input laser beam coupling conditions. Experiments performed by the teams of S. Wabnitz, V. Couderc, and M. Papi have shown that self-cleaning can lead to a significant improvement in the resolution of multispectral multiphoton fluorescence and M-CARS images. This improvement is observed in both microscopy and endoscopy when using GRIN MM fibers to deliver the laser beam to the specimen/sample [47,137,146]. To evaluate the advantages of the described imaging system, 2D and 3D spatial resolutions were determined. For example, a “clean” beam at a 1064 nm wavelength provides a transverse and longitudinal resolution of 0.66 µm and 3.1 µm, respectively. This represents a significant improvement over the linear MM mode of operation, in which multiple focal points caused by the chaotic shape of the initial speckle beam significantly degrade spatial resolution beyond 1.5 µm.

**Figure 6:**
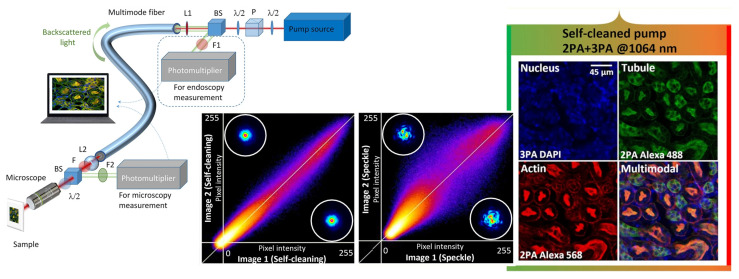
**Left** panel—diagram of beam delivery to the image via multimode fiber; **center** panel—demonstration of stable imaging as a result of Kerr self-cleaning compared to a speckle beam at the exit of a gradient waveguide; **right** panel—images of the kidney stained with Alexa 488, Alexa 568, and DAPI to highlight tubules, actin, and nucleus, respectively, and visualized with a multimode self-cleaned beam (adopted from Moussa et al. under CC-BY 4.0 license [137]).

The most important feature of this imaging system is the reliability of images obtained using “clean” beams compared to speckle beams. Thus, the central part of Figure 6 demonstrates the spatial correlation between numerous images obtained after exposure of the MM fiber to various external disturbances (for example, tension or bending). Images captured in self-cleaning mode remain stable and unaffected by environmental factors. In contrast, images obtained with speckle beams (i.e., beam powers below the self-cleaning threshold) exhibit high instability and are prone to blurring. Due to the advantages listed for the system, virtually all of the innovative in vivo research methods based on multiphoton microscopy that are currently being developed in dermatology will also become available for endoscopic research. In particular, most of the traditional histological procedures can be replaced by morphological and structural analysis of tissues [147]. This will enable visualization and quantification of drug delivery, cancer detection, and investigation of collagen structural transitions.

### 6.2. Numerical Modeling

Numerical modeling is indispensable for understanding the nonlinear beam dynamics in multimode fibers as it not only validates experimental observations but also provides a framework for optimizing system parameters and exploring regimes inaccessible in laboratory settings. Simulation provides insight into the complex interplay of modal dispersion, Kerr nonlinearity, and linear random coupling. In recent years, various mathematical models have been proposed to describe these processes, ranging from simplified approximations to full-scale numerical models.

A foundational tool for both beam self-cleaning and supercontinuum generation (SCG) studies is the generalized 3D nonlinear Schrödinger Equation (GNLSE3D). This full spatiotemporal model captures transverse mode beating, Kerr nonlinearity, linear modal dispersion, and self-imaging in MM GRIN fibers. Using this framework, in [136], Kerr beam self-cleaning was reproduced, and it was shown that the nonlinear energy flow leads to the dominance of the fundamental mode with reduced higher-order contributions. The same model was applied to broadband SCG, where it reproduced geometric parametric instability, dispersive-wave emission, and octave-spanning spectra in both silica and non-silica GRIN fibers [148,149]. Although GNLSE3D is highly accurate, its computational cost scales unfavorably with the number of modes, prompting the development of reduced models.

One important class consists of effective 1 + 1D GNLSE models tailored for graded-index fibers. These models incorporate transverse dynamics into a longitudinally periodic or averaged nonlinear Kerr coefficient. The resulting equations retain key features of modal self-imaging while enabling fast simulations. They have successfully reproduced geometric parametric instabilities and broadband dispersive-wave emission in GRIN fibers [150] and have been used to model multimode soliton dynamics, including formation, fission, and dispersive-wave shedding, which seed SCG [151]. Extensions including Raman response, higher-order dispersion, and self-steepening enable quantitative agreement with experiments on high-power, cascaded-Raman-pumped SC sources in GRIN fibers [152].

For a more explicit description of intermodal coupling, a multimode GNLSE (MM-GNLSE) has been developed. This formalism describes the evolution of each guided mode and incorporates mode-dependent dispersion, Kerr and Raman nonlinearities, self-steepening, and wavelength-dependent intermodal coupling tensors [153]. The MM-GNLSE has been applied to SCG in step-index and graded-index MMFs [154], revealing how intermodal four-wave mixing and spatiotemporal oscillations shape the supercontinuum [155]. Reformulation into ordinary differential equations in the spectral domain [156] and the development of GPU-accelerated solvers [157,158] show efficiency, enabling simulations involving dozens of modes over long fiber spans. The same framework has also been used to model mode-selective SCG in few-mode liquid-core fibers, showing dual dispersive-wave generation and intermodal XPM-induced radiation in agreement with measurements [159].

When the number of modes becomes very large, full-field (3 + 1)D spatiotemporal simulations offer an alternative to modal decompositions. These models directly evolve the field in the transverse coordinates and propagation direction, bypassing mode expansion and outperforming modal models when hundreds of modes are excited. They have produced quantitative agreement with broadband SCG spectra in GRIN fibers [149], as well as in tellurite and chalcogenide multimode platforms, capturing both multimode soliton dynamics and high-order dispersive-wave emission [160].

In parallel, simplified coupled-mode models provide a complementary, physically transparent description of intermodal energy exchange. In [161], such a model was introduced to describe Kerr beam self-cleaning as a turbulence-like cascade involving both direct and inverse energy transfer among modes. Its main advantage was the ability to use large integration steps, making it practical for long-fiber simulations. This model was extended in [162] to incorporate random linear mode coupling arising from fiber imperfections, demonstrating numerically and experimentally that disorder can accelerate beam self-cleaning. A further generalization including Raman gain and intracavity filtering in a multimode fiber laser was developed in [163], explaining the formation of a nearly single-mode Stokes beam through the combined action of Raman gain competition and Kerr-induced modal condensation.

Despite the substantial progress across these modeling approaches, no existing framework fully captures all relevant processes—broadband dispersion, modal disorder, nonlinear gain, vectorial effects, and long-distance propagation—within a single computationally efficient model, leaving substantial room for future refinements.

### 6.3. Image Acquisition Scanning System

The use of laser beams obtained as a result of self-cleaning allows the achievement of the maximum resolution provided by the microscopic system (excluding the MM fiber for beam transport, see ref. [137]), which is 0.37 µm and 0.54 µm in the near infrared and infrared regions of the spectrum, respectively. However, there is a big challenge in miniaturizing the bulky MPM optics into a compact and flexible probe applicable for endoscopic investigations. An analysis of modern developments in piezoelectric scanning multiphoton endomicroscopy, as presented in the review [164], shows that this field has achieved significant technological progress. Systems with various scanning schemes actuated by piezoelectric drives have been implemented as follows: raster (up to 4.1 frames/s) [165], spiral (from 8 to 30 frames/s) [166,167], and Lissajous (up to 5 frames/s) [168]. The key element of these systems is a piezoelectric scanner operating based on the resonant oscillations of a system. Technical solutions vary from the use of piezoelectric tubes [169], bimorphs [165], and four-plate piezoelectric actuators [170] to complex systems with silicon counterweights for resonant frequency separation and reduction in mechanical cross-talk [168]. Typical characteristics of such endoscopes include a high lateral resolution (0.7–0.8 µm) and compact distal probe dimensions (diameter 2–3.5 mm, length 30–53 mm). It should be noted that the achievable scanning ranges (field-of-view) in the described systems vary from 60×60 µm to 450×450 µm, which covers the requirements for both cellular analysis and general visualization of tissue structures.

### 6.4. Artificial Intelligence-Assisted Post-Processing

Multiphoton microscopy methods, due to their extremely high spatial resolution and acquisition speed, allow the accumulation of an enormous volume of morphological and structural information about the tissues being studied. To analyze such a volume, machine learning algorithms are actively used, including the random forest method and the support vector machine. These algorithms have proven themselves well in classification tasks and, when applied in structural analysis, already allow for the rapid identification of disease stages in the tissues studied [171,172]. Thus, the first work presents the results of two-photon in vivo visualization of lymphedema tissue. This study involved 36 image samples from patients with stage II lymphedema and 42 image samples from healthy volunteers. The papillary layer of the skin was examined with a penetration depth of about 100 µm. During this study, both disorganization of the collagen network and an increase in the collagen/elastin ratio in lymphedema tissue were observed, characterizing the severity of fibrosis. Classification using ”ensemble learning” achieved 96% accuracy on the test set [171]. The second work focused on the structure of collagen [172], which is associated with the pathogenesis of many skin diseases. However, collagen also plays an important role in maintaining gut health. It helps restore the mucous membrane of the stomach and intestines, which is important for good digestion and absorption of nutrients.

## 7. Discussion: Fiber-Optic MPEM Application for Optical Biopsy and Digital Histology

In clinical contexts, MPEM is widely being discussed as the perfect perspective for “optical biopsy,” where high-resolution images of tissue histology can be obtained in vivo without the need for any biopsy excision. A major motivation for multiphoton endoscopy is to enable in vivo imaging in situations where WLE is insufficient and CLE is impractical (see Table 2). The main MPEM advantage in comparison with other endoscopic techniques is its ability to diagnose GI diseases via real-time tissue imaging with subcellular detail comparable to CLE histology without the need for staining or contrast infusion [173,174]. To date, MPEM is considered for GI diagnostics applications by visualizing tissue structure (collagen SHG) and metabolic states (redox ratio), enabling differentiation of normal vs. inflamed/neoplastic tissues, assessing fibrosis, and monitoring CRC therapy and GI wound healing (see Table 1). Beyond in vivo diagnostics, fiber-coupled multiphoton microscopy is proving to be a valuable basis for ex vivo tissue analysis, referred to as “digital histology.” As MPEM can generate images based on endogenous fluorescent molecules and second harmonic signals, it can produce label-free contrasts analogous to common stains (for example, SHG highlights collagen like a trichrome stain, and two-photon autofluorescence reveals cellular metabolite distribution similar to cytoplasmic staining). A 2023 study by Galli et al. demonstrates the capability of label-free multiphoton microscopy for histopathological assessment [173]. This research examined cryo-conserved resected human liver tissues containing colorectal cancer metastases using a multimodal MPM setup (combining three endogenous signals: CARS for lipids, TPEF for autofluorescent cell components, and SHG for collagen). Over 40,000 images from 106 patients were acquired, and this morpho-chemical information allowed us to clearly distinguish healthy liver parenchyma from tumors and to identify tumor margins on both frozen sections and unsectioned tissue. The obtained MPM images gave sufficient detail to detect glandular structures, nuclear crowding, and collagen deposition, corresponding to features in conventional histology. Using texture analysis, the authors achieved 95% accuracy in automated classification of tumor vs. normal tissue based on the label-free images [173]. This gives approval for MPEM’s potential application in GI surgery, which would be to scan the excised tissue or the resection bed with a fiber-delivered MPEM device and get immediate feedback on whether margins are clear instead of waiting a minimum of 30 min for the fastest histopathology analysis.

Research in this area has yielded several fiber-optic multiphoton designs. Dilipkumar et al. reported a compact MPM endoscope for in vivo imaging in murine GI in a mouse model of acute intestinal inflammation using a side-viewing GRIN needle lens combined with a femtosecond fiber laser, which allowed repeated imaging in the same animal in order to study the process of inflammation at the tissue level within a period of ten days [175]. This system demonstrated capturing autofluorescence and SHG signals from colonic mucosa, enabling in vivo “histopathology” of experimental colitis progression without any dyes in mice. Another study oriented for quantification assessed collagen-I, NADH, and FAD in two experimental murine colitis models (DSS-induced (20 animals) and CD4^+^CD25^–^ T-cell-transfer (7 animals)) in vivo and ex vivo under conventional histological control, presenting precise evaluation and grading of the disease with a three-channel multiphoton endomicroscope in combination with a novel multiphoton colitis score [176]. Another recent study combined CARS, SHG, and TPEF imaging in a 2.4 mm diameter tip by using a resonant fiber scanner [177]. The authors utilized a special double-core fiber to deliver two synchronized femtosecond pulse trains (for CARS) with low loss and a double-clad structure to return multimodal signals efficiently. The result was a label-free endoscopic microscope that achieved submicron resolution and captured chemically specific images (via CARS) along with structural SHG and fluorescence at about 1 frame/s. The high 0.55 NA distal optics and fiber scanner enabled true histology-like images through a minimally invasive probe. Importantly, this technology is aimed at routine medical use for in vivo nonlinear endoscopy [177]. Progress is also being made on lensless fiber-bundle endoscopes using multicore fibers and distal wavefront control [178]. Another small-sized and cost-effective fiber-optic two-photon endomicroscope demonstrated high detection sensitivity and is capable of capturing subcellular-level label-free tissue images in real time (∼3 fps), assessing structural and dynamic functional/metabolic information in vivo with a spatial resolution and image quality approaching a standard bench-top two-photon microscope [128]. Clinically, one of the earliest adopters of in vivo multiphoton “optical biopsies” was dermatology. Multiphoton tomography devices (e.g., JenLab’s MPTflex) deliver a tunable Ti:Sapphire laser through a flexible articulated arm to a handheld objective for skin imaging (jenlab.dejenlab.de). The MPTflex is a CE-certified clinical system that uses femtosecond excitation in the range of 710–920 nm to capture autofluorescence from NAD(P)H, FAD, melanin, etc., as well as SHG from collagen in intact skin. It provides submicron resolution images down to about a 200 µm depth in seconds, allowing non-invasive diagnosis of skin lesions like melanoma by visualizing cell morphology in vivo. This exemplifies how fiber-/arm-delivered multiphoton imaging is already being translated to clinical practice for in vivo histology of accessible tissues (skin), and ongoing research aims to extend the technology to internal organs via endoscopic approaches.

Beyond in vivo diagnostics, fiber-coupled multiphoton microscopy is proving valuable for ex vivo tissue analysis, called “digital histology.” Another key question posed was whether MPM can be used for label-free histology on extracted tissues—comparative studies show that the answer is yes, with results approaching conventional pathology in accuracy. The thorough comparative MPM study by Makino et al. provided sufficient histologic detail to identify all relevant substructures ex vivo in healthy GI tissue, visualize both acute and resolving stages of colitis, and assess the progression of colorectal carcinogenesis in mouse IBD and CRC models. Ex vivo specimens from human subjects with celiac sprue, inflammatory bowel disease, and colorectal neoplasia were examined by MPM within 1 h after biopsy resection. Finally, colonic mucosa in live anesthetized rats was imaged in vivo using a flexible endoscope prototype [179]. In both animal models and also human specimens, the MPM images showed maximal similarity to the results of conventional histological staining, supported by the pathologists’ diagnoses [180].

It should be noted that using MPM for histopathology does not strictly require fiber delivery—many studies use bench-top MPM. However, to integrate this into surgical workflows, fiber-delivered or portable MPEM systems are undoubtedly advantageous. Current MPEM studies are successful in both in vivo and ex vivo approaches involving clinical samples (see Table 3), and, in the latest decade, also in AI-enhanced classification, improving speed and accuracy. However, larger clinical research and standardization of protocols are needed for broad MPEM adoption in GI diagnostics before being established as a standard of care. Although MPEM implementation represents a significant technical challenge and still promises high costs, the attractiveness of the possibilities compared to existing clinical approaches seems to significantly outweigh these limitations.

## 8. Conclusions

This review presents the potential to move MPM optical biopsy from a concept to a widely used technique by summarizing data on current research and innovative technology development that can substantially improve image resolution, contrast, and tissue penetration. It describes modern approaches and achievements in identifying informative features in images of biological tissue collagen obtained by multiphoton microscopy, and on this basis, algorithms for computer classification of its structures are developed. What is noteworthy is that all of these developments became available only recently and can be directly used to create an imaging system to classify gastrointestinal diseases in vivo and determine their stage. This is an extremely challenging task as it requires a strong multidisciplinary background. We believe that this is the direction in which researchers will concentrate their efforts in the next decade. 

## Figures and Tables

**Table 1:** Spectral characteristics of endogenous fluorophores.

Endogenous Fluorophores	Autofluorescence Excitation and Emission Ranges		Two-Photon Excitation Wavelengths	GI Tissue Property Assessment
NAD(P)H	372 nm 375 nm 330–380 nm 330–360 nm	430–480 nm [75] 456–484 nm [76] 440–462 nm [61] 440–470 nm [77]	750 nm (detection with 485 nm band-pass filter) [78]	Redox state of tissue [78]
FAD	438 nm 445 nm 360–465 nm	500–550 nm [75] 456–484 nm [76] 520–530 nm [77]	800 nm (detection with 530 nm band-pass filter) [78]	Redox state of tissue [78]
Vitamin A	370–380 nm	490–510 nm [61,79]	700–1000 nm [80]	Potential marker for CRC [81]
Lipofuscin	400–500 nm 450–490 nm	480–700 nm [61] 400–700 nm [82]	700–850 nm [83]	Potential marker for CRC [81]
Keratin	280–325 nm 355–415 nm 488 nm	495–525 nm [61] 400–520 nm [84] 500–550 nm [85]	730–820 nm [86]	Potential marker for IBD [87]
Collagen	320–410 nm 375 nm	400–510 nm [88] 380–420 nm [76]	800–820 nm (SHG) [89]	Stromal remodeling [90]

**Table 2:** Comparison of optical assessment modalities in GI endoscopy.

	WLE	NBI	CLE	M-CARS	MPEM
Assessment mode	In vivo	In vivo	In vivo/ex vivo/ probe-based	In vivo/ex vivo	In vivo/ex vivo/ probe-based
Resolution	Low	Low	High	High	High
Field of view	cm^2^s; 90°–170°	2–100 mm; 140°–170°	0.1–0.3 mm	0.3–0.5 mm	0.3–0.5 mm
Frame rate (Speed)	30–60 fps	30–60 fps	<1.6 fps	1.4–20 fps	4–10 fps
Depth	Surface-level only	<200 µm	0–100 µm, limited to focal plane	20–100 µm	200–400 µm, 3D imaging
Need for labeling or contrast agent	None	None (Optical filter)	Fluorescent dyes required	None (Molecular fingerprint)	None (Autofluorescence based)
Detection specificity	Visual inflammation, tissue lesions	Vascular and mucosal patterns	Pathology-specific staining	Lipid, protein, chromatin	Lipid, protein, oxidative status, inflammation, stromal remodeling
Sensitivity	Low	High	High	High	High
Diagnostic efficacy	Low	High	High	High	High
Clinical status	Standard of care	Established standard of care	Established for targeted use	Experimental, Pre-clinical	Experimental, Pre-clinical
Limitations	Low resolution and diagnostic efficacy; need for endoscopist’s experience	Restrictions in diagnostics; need for endoscopist’s experience	Need for labeling or contrast agent;time consumption	Technical complexity; high cost; complex data interpretation	Technical complexity; high cost

**Table 3:** Summary of GI MPEM studies (in vivo and ex vivo).

Study Focus	Assessment Mode, Sample Origin	Sample Details	Diagnostic Endpoint
Mucosal histology	Ex vivo (Human), in vivo (Rat)	Fresh human GI biopsies; intact rat colon	Label-free identification of epithelial nuclei, goblet cells, and fibers [180]
IBD	In vivo (Mouse), ex vivo (Mouse)	Live mouse GI tract of the experimental colitis models; fresh colon tissue from experimental colitis models	Quantification of inflammatory tissue remodeling; identification of model-specific disease patterns [175,176]
Gastrointestinal carcinogenesis progression	Ex vivo (Human), ex vivo (Mouse), in vivo (Rat), in vivo (Mouse)	Fresh biopsies of human celiac sprue, IBD, and neoplasia; live rat colonic mucosa; live mouse GI tract	Identification of colitis stages (acute/resolving) and progression of colorectal carcinogenesis; visual detail comparable to conventional histology [175,176,180]
CRC	Ex vivo (Human)	30 patients (CRC biopsies); 7 patients (colorectal carcinoma)	High diagnostic accuracy (93.5–99.0%) for invasion depth and histologic classification [89,181]
Organ microstructure	In vivo (Rat), ex vivo (Human), ex vivo (Porcine)	Live rat liver, kidney, and colon; human intestinal wall; porcine intestinal wall	Real-time imaging using 800 nm light at 4.1 fps; morphological features 75% consistency with conventional histology [178,182]

## Data Availability

No new data were created or analyzed in this study. Data sharing is not applicable to this article.
